# *Tilapia nilotica* Head Lipids Improved Bone Loss by Regulating Inflammation and Serum Metabolism Through Gut Microbiota in Ovariectomized Rats

**DOI:** 10.3389/fnut.2021.792793

**Published:** 2022-01-12

**Authors:** Yujie Zhu, Shucheng Liu, Fengfeng Mei, Meihui Zhao, Guanghua Xia, Xuanri Shen

**Affiliations:** ^1^Key Laboratory of Food Nutrition and Functional Food of Hainan Province, Key Laboratory of Seafood Processing of Haikou, Hainan Engineering Research Center of Aquatic Resources Efficient Utilization in South China Sea, College of Food Science and Technology, Hainan University, Hainan, China; ^2^Guangdong Provincial Key Laboratory of Aquatic Product Processing and Safety, Guangdong Ocean University, Zhanjiang, China; ^3^Collaborative Innovation Center of Provincial and Ministerial Co-construction for Marine Food Deep Processing, Dalian Polytechnic University, Dalian, China

**Keywords:** bone loss, *Tilapia nilotica* head lipids, gut microbiota, inflammation, metabonomics

## Abstract

Osteoporosis is a global health problem, and it is of great significance to replace the drugs with natural functional factors. In this study, we investigated the antiosteoporotic activity of lipids prepared from *Tilapia nilotica* fish head lipids (THLs) in the ovariectomized osteoporosis rats. THLs are composed of neutral lipids (NL, 77.84%), phospholipids (PL, 11.86%), and glycolipids (GL, 6.47%). There were apparent differences in the fatty acid composition of disparate components, and PL contains the most abundant Ω-3 polyunsaturated fatty acids. The results proved that THLs could improve bone microstructure, increase bone mineral density, and decrease bone resorption. To illustrate the antiosteoporotic mechanism, we analyzed the changes in gut microbial communities, proinflammation factors, serum metabolites, and metabolic pathways. Further study on gut microbiota showed that THLs significantly decreased the content of *Alistipes* in the gut and dramatically increased the beneficial bacteria such as *Oscillospira, Roseburia*, and *Dubosiella*. Meanwhile, proinflammation factors of serum in OVX rats decreased significantly, and metabolites were changed. Therefore, we speculated that THLs improved bone loss through reducing inflammation and changing the metabolites and metabolic pathways such as arachidonic acid metabolism and primary bile acid metabolism, etc., by altering gut microbiota. The results indicated that THLs could be a functional factor with antiosteoporotic activity.

## Introduction

Bone is a dynamic tissue continuously updated through absorption of old bone and formation of new bone (1). To achieve and maintain the bones' size, shape, and structural integrity, vertebrates continue to remodel their bones in adulthood, remove, and repair damaged bones (2). The imbalance of bone remodeling results in severe bone loss and bone quality decline, which results in all kinds of bone diseases, such as rheumatoid arthritis, osteoporosis, and deformity osteoarthritis (3). Osteoporosis occurs when bone resorption mediated by osteoclasts greater than bone formation regulated by osteoblasts, which is defined by international consensus as a systematic skeletal disease characterized by a decrease in bone mineral density and damage of bone microstructure, which generates increased bone vulnerability and fracture susceptibility (4–6). Women are more likely to contract osteoporosis after menopause, because estrogen is a vital regulatory factor of bone homeostasis (7–9). Estrogen deficiency can lead to faster bone turnover and raise the threat of fractures (10). However, the risks of estrogen therapy for older women are more significant than its benefits, so it is not recommended (11). In addition to estrogen, alendronate, calcitonin, raloxifene, sodium fluoride, calcitriol, and diphosphate have also been used to treat osteoporosis (12). However, osteoporosis needs a long-term treatment period, and these medicines will cause urinary calculi, endometrial cancer, breast cancer, and other side effects (13). Therefore, there is an urgent need for secure and effective functional factors to the prevent and treat osteoporosis.

It is well-known that bone formation and bone resorption are critical process of bone remodeling. During bone formation, preosteoblasts stemmed from bone marrow mesenchymal stem cells, undergo proliferation, differentiation, and mineralization to form mature osteoblasts (14). The decrease of mature osteoblasts can cause an imbalance of bone metabolism and bone loss. Another essential factor that causes the imbalance of bone metabolism is the excessive increase of bone resorption mediated by osteoclasts. When bone resorption is stronger than bone formation, it will cause bone loss and bone microstructure damage and then result in osteoporosis.

Gut microbiota is a symbiotic microorganism that lines in the gut and is more than the number of all human cells (15, 16). Gut microbiota principally includes *Bacteroides, Firmicutes, Actinomycetes, Proteobacteria, Verrucomicrobes*, and other microbial groups (17). In healthy individuals, microbial community, host, and environment are always in a steady dynamic equilibrium. Plenty of evidences (18–23) indicated that gut microbiota plays a crucial role in adjusting bone metabolism. According to reports, the gut microbiota interplays with the host to regulate the intestinal and affect immune cells by producing molecules with immunomodulatory and antiinflammatory functions, thereby controlling the immune system to affect bone metabolism (21, 24). Many studies have proved that probiotics in gut microbiota such as *Lactobacillus reuter, Lactobacillus rhamnosua*, and *Bifidobacterium Longum* can inhibit intestinal inflammation, suppress the generation of osteoclasts, and reduce bone loss (25–27). Gut microbiota also affects metabolites, and the decrease of the proportion of *Bacteroides* or *Firmicum* will lead to the disorder of host metabolism and destroy bone homeostasis (22). Metabolomics is used to comprehensively explore the change of metabolite content in biological samples to understand the mechanism of disease, which is sensitive and unbiased (28). For the past few years, metabonomics has been widely used in the studied of bone-related diseases such as osteonecrosis (29), osteoarthritis (30), and osteoporosis (31).

*Tilapia nilotica* is one of the most important farmed fishes and has been commercialized in many countries. China is the largest producer and consumer of *Tilapia nilotica* (32). The fish head is one of the primary by-products produced during the processing of *Tilapia nilotica. Tilapia nilotica* fish head lipids (THLs) are rich in glycolipids, phospholipids, and other active ingredients. It has been found that marine lipids rich in EPA can protect against bone loss (33). Our previous research found that THLs can promote cell proliferation of MC3T3-E1 cells and proved that it has a significant antiinflammatory effect (34). As the improvement effect of THLs on bone loss in ovariectomized rats was proved, its relationship with gut microbiota and metabolites remains unclear.

In this experiment, we analyzed the composition of THLs, which investigated its antiosteoporotic activity in ovariectomized osteoporosis rats and explored its antiosteoporotic mechanism from the aspects of gut microbiota and serum metabolism, hoping to promote effective utilization of by-products *Tilapia nilotica*.

## Materials and Methods

### Materials and Reagents

*Tilapia nilotica* heads were purchased from Xiangtai Fishery (Hainan, China) and were transferred to the laboratory below 4°C at once. Enzyme-linked immunosorbent assay (ELISA) kits were obtained from Shanghai Enzyme-linked Biotechnology Limited Company (Shanghai, China).

### Preparation of THLs

The preparation method of THLs was based on Chen et al. (35) and modified slightly. The *Tilapia nilotica* heads were cleaned with water to get rid of the gills and other impurities, crushed by an ultrafine pulverizer, centrifuged to remove water, and proceeded to the next experiment. A certain volume of 95% ethanol was added in the ratio of 1:4 (w/v), ultrasonically extracted for 1.5 h at 25°C, and repeated the extraction three times. The resulting ethanol extract was concentrated by rotary evaporation at 45°C to obtain the crude THLs.

The crude ethanol extract was mixed with chloroform or methanol or water at the ratio of 8:4:3 (v/v/v) in the centrifugal tube. The mixture was vortexed for 1 min to remove impurities such as pigments and then centrifuged for 10 min at 25°C and 4,000 g. The lower organic layer was collected, added 0.2 times the volume of 0.9% (m/v) NaCl solution, vortexed for 1 min to remove the protein, centrifuged again under the above conditions, collected the lower organic layer, and concentrated it on a rotary evaporator to obtain the purification THLs.

### Composition and Fatty Acid Analysis of THLs

*Tilapia nilotica* fish head lipids were separated using the method of medium-pressure purification in a medium-pressure preparation and purification apparatus. THLs were isolated by silica gel column chromatography, and each component was eluted with chloroform, acetone, and methanol in turn. In the process of separation, chloroform, acetone, and methanol were used as eluents to obtain fr I (neutral lipid, NL), fr II (glycolipid, GL), and fr III (phospholipid, PL).

The fatty acid composition was analyzed by Agilent 6890N gas chromatograph. The percentage of fatty acid was represented as the peak area percentage and determined by the area normalization method. After methylation, fatty acid methyl esters are identified (36). The composition and percentage of fatty acids in THLs, NL, GL, and PL are computed by comparing the holding time of the chromatographic peaks of standard samples of fatty acid methyl esters.

### Animal Procedures and OVX Osteoporosis Rat Model

Three-month-old female pure Wistar rats were supplied by Tianqin Biotechnology Company of Changsha. Rats were moved freely and provided food and water in the cages. Cages were kept at 23 ± 1°C and 56 ± 4% relative humidity, and the period of light and shade is 12 h. All animal procedures were operated following the Guidelines for Care and Use of Laboratory Animals of Hainan University and permitted by the Animal Ethics Committee of Hainan University.

Adaptive feeding continued for 1 week, and then, the rats were split into five groups (*n* = 8) at random: the normal control group (Sham operation group, marked as “Sham”), OVX model group (marked as “OVX”), OVX-positive estrogen drug group (marked as “E2”), OVX THLs high-dose group (marked as “Tn-H”), and OVX THLs low-dose group (marked as “Tn-L”). Rats were performed bilateral laparotomy (Sham, *n* = 8) or bilateral ovariectomy (OVX, *n* = 32) under anesthesia with 0.25 mL/100 gab 10% chloral hydrate. After surgery, 20,000 units of ampicillin sodium were injected into the limb muscles for three consecutive days. The rats were fed normally for 30 days. Then, the rats in each group were given corresponding gavages. Gavages were given once a day at a fixed time for 90 days.

The volume of the test substance or normal saline was 1 mL/100 g.bw. Rats of Sham and OVX groups were orally administered normal saline, rats in group Tn-L were orally administered 200 mg/kg.bw THLs, those in group Tn-H were orally administered 400 mg/kg.bw THLs, and rats in group E2 were orally administered 300 mg/kg.bw estradiol tablets. After intragastric administration for 90 days, 0.25 mL/100 g.bw 10% chloral hydrate was intraperitoneally injected, and the rats were sacrificed (37, 38).

### Micro-CT Bone Analysis

After the rats were sacrificed, to estimate the loss extent of bone structure, the femora of the right leg of the rats was quickly removed, and the far end of the femora was fixed in neutral methanol, washed in running water, and stored in 75% ethanol. The samples were scanned with microcomputed tomography (Micro-CT). Scanning parameters were voltage 70 kV, current 114 μA, and scanning resolution 10 μm.

### Analysis of Serum Biochemical Indexes and Proinflammatory Cytokine

At the end of the dissection, whole blood samples were gathered from the abdominal vein. Blood was clotted for 30 min and then was centrifugated at 3,000 g for 10 min to separate the serum. The levels of blood biochemical index and proinflammatory cytokine were determined with ELISA kits for tartrate resistant acid phosphatase 5β (TRAP-5β), crosslinked C-terminal peptide of type I collagen (CTX-1), cathepsin K (Cath-K), matrix metalloproteinase 9 (MMP-9), osteoprotegerin (OPG), receptor activator of the NF-κB ligand (RANKL), tumor necrosis factor-α (TNF-α), interleukin 6 (IL-6), interleukin 17 (IL-17), and macrophage colony-stimulating factor (M-CSF). All ELISA operation steps were performed based on the manufacturer's instructions (39).

### 16S rDNA High-Throughput Sequencing

Fecal samples were collected before the animals were sacrificed for gut microbiota analysis. The gut bacterial composition of rats in each group was analyzed through 16S rDNA gene analysis. The extraction method of genomic DNA of the sample was the CTAB method, and the detection method of purity and concentration of the DNA was agarose gel electrophoresis. A suitable amount of the sample DNA was got out from a centrifuge tube, and the sample was diluted to 1 ng/μL with sterile water. We selected one or several variant regions, used conservative regions to design universal primers for PCR amplification, and then performed sequencing analysis and bacterial strain identification on the hypervariable regions. QIIME analysis software (version 1.9.1) and R analysis software (version 2.15.3) were used for alpha diversity analysis and beta diversity analysis to show the richness and diversity of microbial communities in the samples and compare the microbial community composition of different samples.

### Serum Metabolites Extraction and LC-MS/MS Analysis

One hundred microliter of serum sample and 400 μL of pre-chilled methanol were mixed by well vortexing, placed on ice for 5 min, and then centrifuged at 15,000 rpm, 4°C for 5 min. The supernatant was injected into the LC-MS/MS system analysis.

The separation was proceeded on a hyposil gold column (C18); column temperature: 40°C, flow rate: 0.2 mL/min, mobile phase A: 0.1% formic acid, and mobile phase B: methanol. The conditions of the ESI source were as follows: spray voltage: 3.2 kV; sheath gas flow rate: 40 arb; aux gas flow rate: 10 arb; capillary temp: 320°C; polarity: positive, negative. MS/MS secondary scans are data-dependent scans. The mass range was recorded at M/Z 70–1,050.

### Metabolites Data Analysis

Raw data files were imported into CD search library software for simple screening of parameters such as retention time and mass charge ratio, and then, the peak alignment of different samples was performed in light of the mass deviation of 5 ppm and the retention time deviation of 0.2 min to ensure the identification more accurate. Perform peak extraction was based on the set mass deviation of 5 ppm, signal intensity deviation of 30%, signal-to-noise ratio 3, the minimum signal intensity of 1,00,000, and sum ions and other information, and peak area for quantitative, to integrate target ion. Then, fragment ions and molecular ion peaks are used to forecast the molecular formula and compare with mzCloud (https://www.mzcloud.org/), mzVault, and Masslist databases. Background ions were removed through using blank samples and normalized the quantitative results. In the end, the identification and quantitative results of the data are obtained.

### Statistical Analysis

Data are shown as mean ± standard deviation (SD). Groups were compared using one-way ANOVA in the SPSS 26.0 software (IBM, Armonk, NY, USA). Graphs were depicted by GraphPad Prism v. 8 (GraphPad Software, San Diego, CA, USA). For all analysis results, *p-*values below 0.05 and 0.01 were considered as significant and extremely significant differences, respectively.

## Results

### Lipid Distribution of THLs and Fatty Acid Composition of THLs, NL, GL, and PL

The yield of THLs was 3.4% (calculated by wet weight). The proportions of the lipid classes are presented in Table 1, the content of NL (77.84%) is the most in THLs, followed by PL (11.86%), and GL (6.47%) is the least.

**Table 1:** Composition (%) of the lipid classes^a^.

**Lipid classes**	**Neutral lipids (NL)**	**Glycolipids (GL)**	**Phospholipids (PL)**
Composition	77.84 ± 2.71	6.47 ± 1.65	11.86 ± 0.23

a *Values are the mean of triplicate analyses ± SD*.

The fatty acid composition and content analysis of THLs, TH-NL, TH-GL, and TH-PL are shown in Table 2. THLs contained 18 kinds of fatty acids, and the number of carbon atoms was between 12 and 22. Saturated fatty acids (SFAs) account for about 35% of the total fatty acid content, mainly C16:0, followed by C14:0 and C18:0; monounsaturated fatty acids (MUFAs) accounted for about 38%, with C18:1 being the most abundant, followed by C16:1. In addition, THLs were also rich in polyunsaturated fatty acids (PUFAs), accounting for about 22.56% of the total fatty acids, of which C18:2 had the highest content, followed by C18:3 n-3 and C20:2 n-6. The total proportion of EPA and DHA is 0.74%.

**Table 2:** Fatty acid composition (%) of TL, NL, GL, and PL^a^.

**Fatty acid**	**THLs**	**TH-NL**	**TH-GL**	**TH-PL**
C12:0	0.10 ± 0.06	0.12 ± 0.09	0.10 ± 0.32	–
C14:0	3.38 ± 0.06	3.50 ± 0.08	2.93 ± 0.17	1.07 ± 0.17
C15:0	0.39 ± 0.09	0.40 ± 0.11	0.36 ± 0.37	0.40 ± 0.41
C16:0	23.83 ± 0.03	23.85 ± 0.07	22.67 ± 0.07	25.45 ± 0.0
C17:0	0.41 ± 0.03	0.41 ± 0.19	0.51 ± 0.16	0.66 ± 0.33
C18:0	6.58 ± 0.43	6.67 ± 0.10	10.26 ± 0.07	11.71 ± 0.0
C20:0	0.32 ± 0.41	0.36 ± 0.22	0.47 ± 0.23	0.66 ± 0.08
∑SFAs	35	35.3	37.31	39.9
${\text{C}}_{16:1}^{\text{Δ}t}$	0.62 ± 0.08	0.48 ± 0.05	0.54 ± 0.06	0.55 ± 0.08
${\text{C}}_{16:1}^{\text{Δ}c}$	5.09 ± 0.01	5.13 ± 0.17	3.94 ± 0.16	1.66 ± 0.30
C17:1	0.24 ± 0.08	0.25 ± 0.66	0.23 ± 0.13	0.21 ± 0.04
C18:1	31.99 ± 0.08	31.60 ± 0.03	30.06 ± 0.27	22.25 ± 0.47
∑ MUFAs	38	37.5	34.76	24.7
C18:2	16.88 ± 0.03	16.90 ± 0.22	14.73 ± 0.43	9.43 ± 0.02
C18:3 n-3	3.28 ± 0.08	3.28 ± 0.04	2.80 ± 0.07	1.27 ± 0.01
C20:2 n-6	0.72 ± 0.43	0.71 ± 0.32	0.76 ± 0.03	0.86 ± 0.09
C20:5 n-3 (EPA)	0.11 ± 0.01	0.18 ± 0.04	0.14 ± 0.09	0.44 ± 0.00
C22:5 n-3 (DPA)	0.33 ± 0.07	0.33 ± 0.08	0.50 ± 0.33	1.07 ± 0.03
C22:5 n-6	0.61 ± 0.04	0.56 ± 0.16	1.33 ± 0.19	3.53 ± 0.06
C22:6 n-3 (DHA)	0.63 ± 0.39	0.57 ± 0.27	1.81 ± 0.22	5.17 ± 0.09
EPA+DHA	0.74	0.75	1.95	5.61
∑ PUFAs	22.56	22.53	22.06	21.77
Other	4.48	4.64	5.87	13.59

a *Values are the mean of triplicate analyses ± SD*.

The content of SFA in NL, GL, and PL was higher than that of MUFA. In terms of SFA, the content of C16:0 was the most abundant in the fatty acid composition of different polar lipids (NL, GL, and PL), accounting for 23.85, 22.67, and 25.45% of the total fatty acid content, respectively; he contents of MUFA in NL, GL, PL were 37.5, 34.76, and 24.7%, respectively. Among them, the highest content of MUFA was fatty acid C18:1. As for PUFA, PL contained the most amount of Ω-3 polyunsaturated fatty acids (6.68%), followed by GL (2.45%) and NL (1.08%).

### THLs Improved Bone Microstructure in the OVX Rats

**Figure 1:**
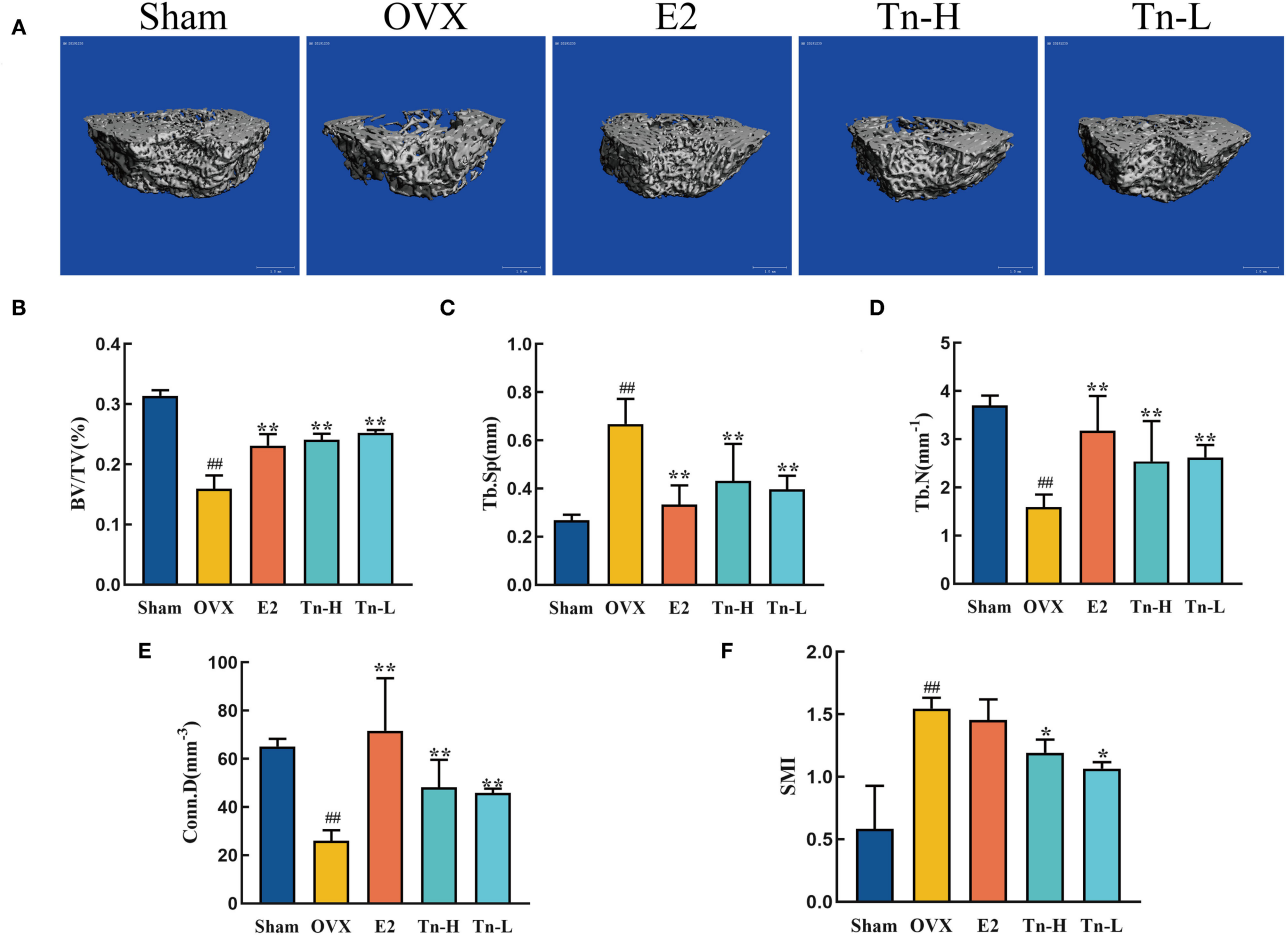
Effect of THLs on the femoral microstructure of ovariectomized rats: **(A)** Transverse views of bone tissue microstructure. **(B–F)** Quantitative results of bone volume fraction (BV/TV), trabecular number (Tb.N), trabecular separation (Tb.Sp), cortical density (Conn.D), and structural model index (SMI) (Tn-H, high-dose THLs group; Tn-L, low -dose THLs group). Data are presented as the mean ± SD (*n* = 8 per group). Multiple comparisons were performed using one-way ANOVA. ^#^*p* < 0.05 vs. Sham group; ^*##*^*p* < 0.01 vs. Sham group; ^*^p < 0.05 vs. OVX group; ^**^p < 0.01 vs. OVX group.

The microstructure of bone tissue can directly judge the state of bone and predict the severity of bone loss. Therefore, Micro-CT technology was used to scan the bone tissue samples and measure the three-dimensional morphological characteristics of the bone tissue microstructure, including BV/TV, Tb.N, Tb.Sp, Conn.D, and SMI. Results were displayed in Figure 1A, in the Sham group, the number of trabeculae was more, the structure was compact, and the reticular structure was complete; In the OVX group, the number of trabeculae was significantly reduced, the reticular structure was destroyed, and the connectivity was decreased. Figures 1B–F explained above phenomenon in the OVX group: Conn.D, BV/TV, and Tb.N were decreased remarkably, and Tb.Sp and SMI were increased distinctly. The E2 group, the THLs high-, and low-dose groups significantly improved trabecular microstructure, which reflected in the significant increase of Conn.D, BV/TV, and Tb.N and the decrease of Tb.Sp and SMI.

### THLs Decreased the Bone Resorption in the OVX Rats

**Figure 2:**
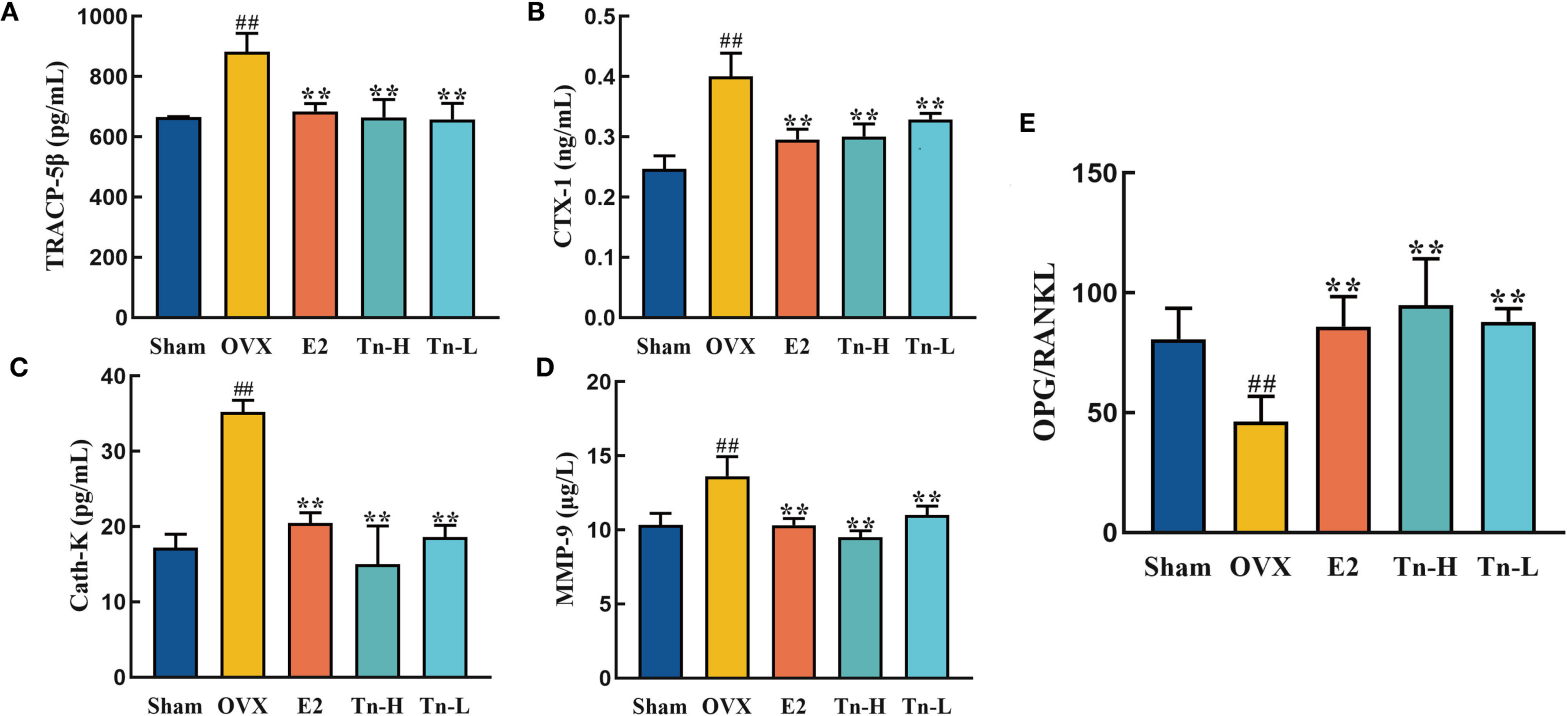
Effect of THLs on bone resorption-related indexes in serum of rats. Bone resorption-related indexes including TRACP-5β **(A)**, CTX-1 **(B)**, Cath-K **(C)**, and MMP-9 **(D)**. **(E)** The ratio of OPG to RANKL at serum level (Tn-H, high-dose THLs group; Tn-L, low-dose THLs group). Data are presented as the mean ± SD (*n* = 8 per group). Multiple comparisons were performed using one-way ANOVA. ^#^*p* < 0.05 vs. Sham group; ^*##*^*p* < 0.01 vs. Sham group; ^*^p < 0.05 vs. OVX group; ^**^p < 0.01 vs. OVX group.

Ovariectomized rats lack estrogen, resulting in an imbalance of bone formation and resorption, which then causes bone loss. The levels of bone resorption markers in serum were analyzed by ELISA. Ovariectomy resulted in significant increases of TRACP-5β, CTX-1, Cath-K, and MMP-9 in serum (Figures 2A–D), indicating that the bone resorption rate was speeded up. The level of TRACP-5β in the E2 and THLs groups decreased significantly compared with OVX group and almost returned to the normal level (Figure 2A). Figures 2B–D showed that THLs could significantly inhibit the abnormal increase of CTX-1, Cath-K, and MMP-9 levels. These results suggested that THLs can minimize the rate of bone resorption and improve bone loss. In addition, we measured the ratio of OPG to RANKL at serum. RANKL can stimulate osteoclast differentiation and maturation, OPG can prevent osteoclast formation, and imbalance of OPG/RANKL will lead to excessive bone resorption. Figure 2E showed that OPG/RANKL decreased significantly when estrogen was deficient. Treatment with E2 and THLs restored the imbalance, and the effect of THLs was slightly better than that of E2. In conclusion, after the oophorectomy, estrogen deficiency leads to the increase of osteoclast activity and decrease of osteoblast activity. The above results bore out that THLs can reduce bone loss by regulating the dynamic balance of bone resorption and bone formation.

### THLs Reduced Inflammation and Improved Bone Loss by Altering Gut Microbiota in OVX Rats

To study the effect of THLs on gut microbiota in ovariectomized osteoporosis rats, 16S rDNA high-throughput sequencing was carried out based on the Illumina Novaseq sequencing platform, which revealed the difference between species composition and community structure among samples. The samples of five groups (Sham, OVX, E2, Tn-H, and Tn-L) obtained 587, 552, 638, 586, and 528 operational taxonomic units (OTUs), respectively.

**Figure 3:**
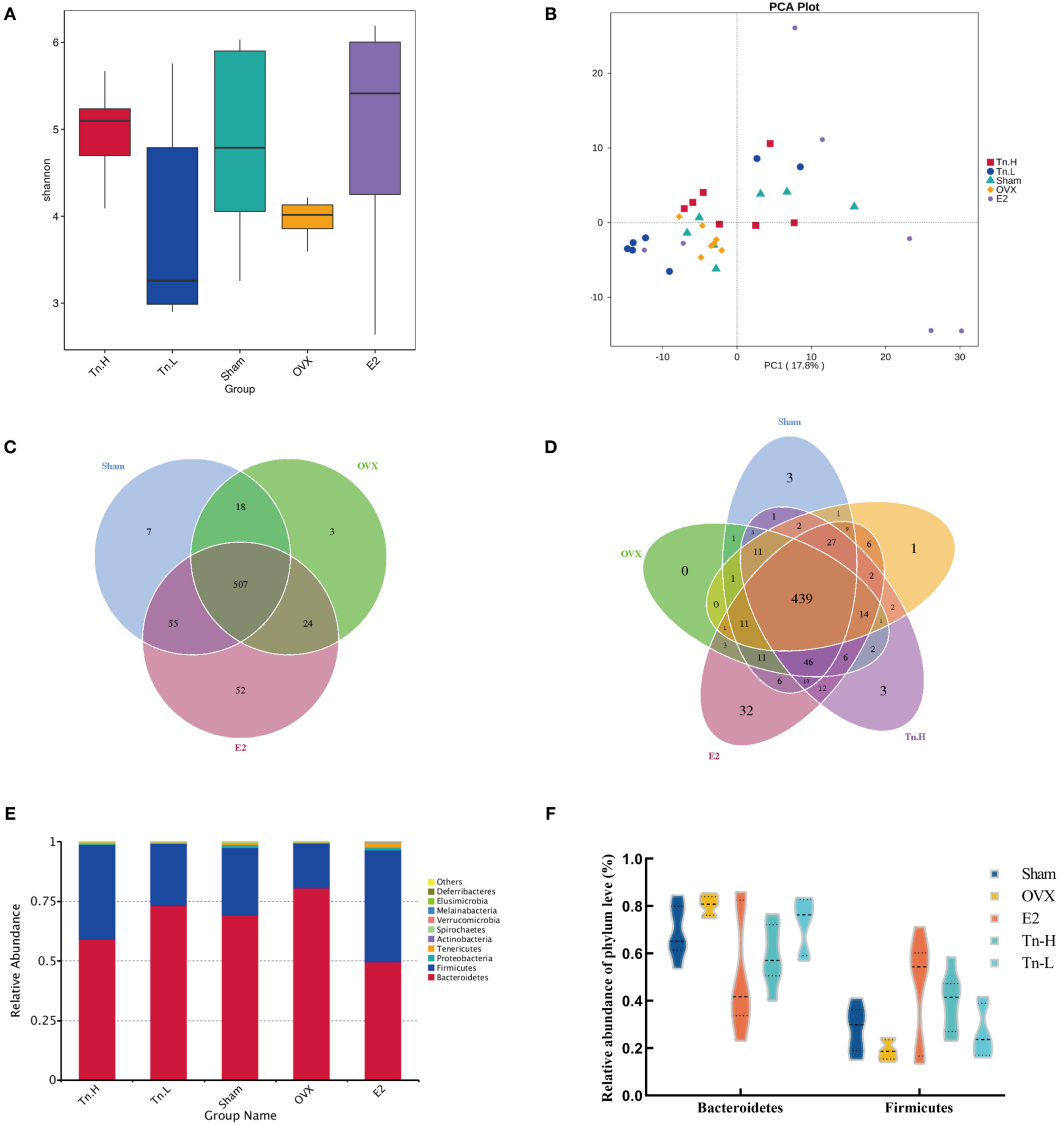
THLs altered gut microbiota in ovariectomized rats. The feces of rats were collected and sequenced, and the community structure was analyzed. Shannon–Wiener species diversity index **(A)** calculated the alpha diversity. PCA **(B)** was performed to show the changes in gut microbiota diversity between groups. The number of common and individual OTUs was displayed in Venn diagram **(C,D)**. Taxonomic classification of the gut microbiome showing the proportion of microorganisms in each group at the phylum level **(E,F)**. *n* = 8 per group, Tn-H, high-dose THLs group; Tn-L, low-dose THLs group.

Alpha diversity was usually used to explain the diversity of microbial communities. Shannon index (Figure 3A), which showed that the microbial complexity of each group, and the Shannon index of OVX group were significantly lower than Sham group. After supplementation of estrogen and high-dose THLs, the microbial richness increased, while low-dose THLs decreased. Beta diversity compared the composition of gut microbiota in different samples. Principal component analysis (PCA) (Figure 3B) based on OTU level showed that there was a significant difference in the composition of gut microbiota between OVX group and Sham, E2 groups. After the treatment of ovariectomized rats with high- and low-dose THLs, the gut microbiota was changed to different extent. The number of shared and unique gut microbiota among different groups was analyzed by drawing Venn graphs (Figures 3C,D). Results suggested that the number of gut microbiota decreased by 62 species and a number of 27 new gut microbiota were produced. The gut microbiota of rats treated with low-dose THLs shared 523 species with OVX group and produced 74 new gut microbiota. The gut microbiota of rats treated with high-dose THLs shared 524 species with OVX group and produced 62 new gut microbiota. The species annotation results found the highest top 10 species with the abundance at the phylum level of each group, and which were picked out to get a chart of species relative abundance. The results showed (Figure 3E) that the most abundant bacteria were *Bacteroidetes, Firmicutes*, and *Proteobacteria* at phylum level. Ovariectomy increased *Bacteroidetes* and decreased *Firmicutes* in rats, which was reversed by the intervention of THLs (Figure 3F).

**Figure 4:**
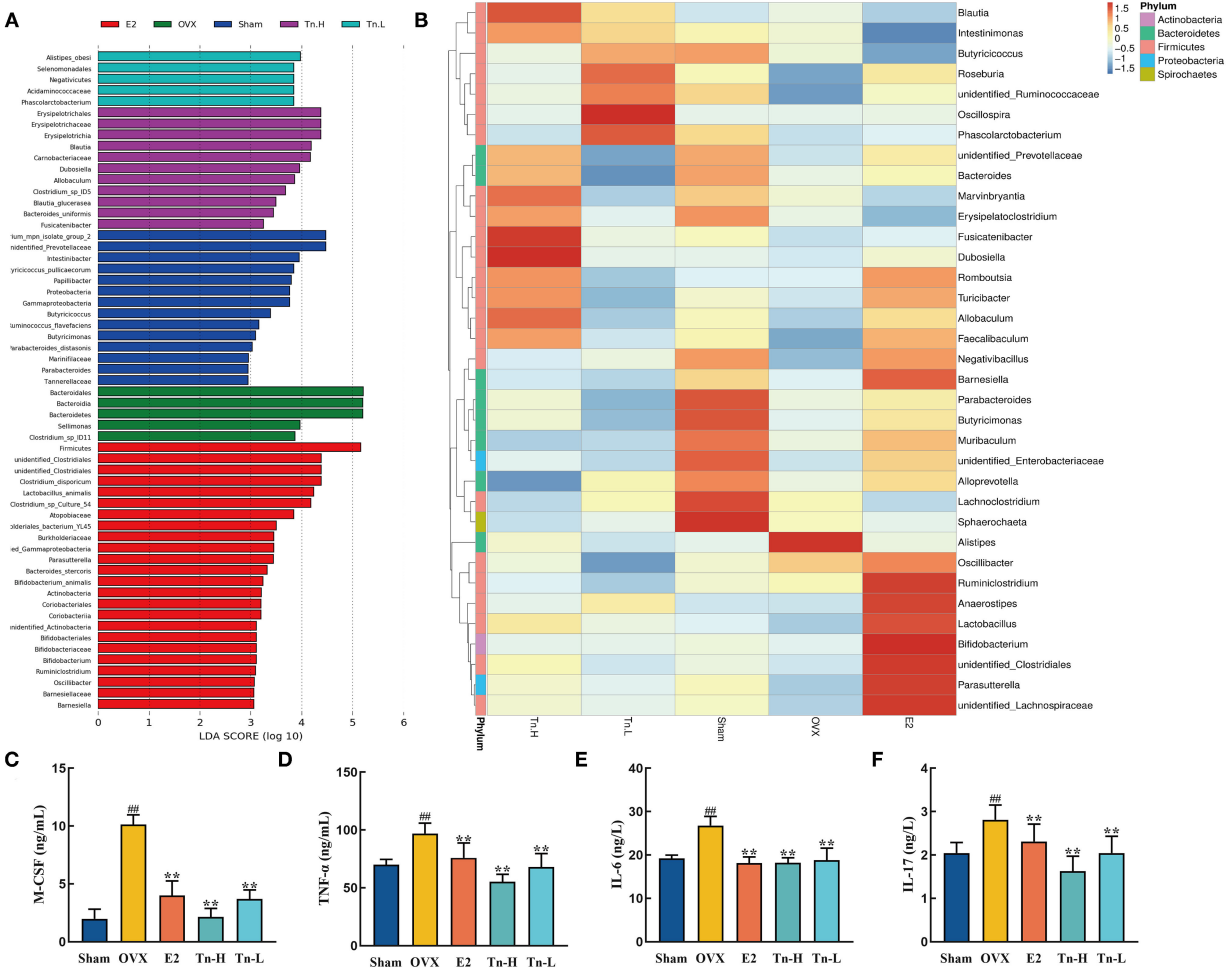
THLs reduced inflammation by altering gut microbiota in OVX rats. LEfSe was applied to analyze the biomarker bacteria for each of groups. LDA score **(A)** is greater than the set value (the default setting is 4), that is, the biomarker with statistical difference between the groups. Species abundance of the genus level was showed in clustering heatmap **(B)**. The abscissa is used to display the names of samples or groups, and the ordinate on the right is used to display the names of bacteria. The deeper the red color, the higher the abundance of the bacteria; the deeper the blue color, the lower the abundance of the bacteria. M-CSF **(C)**, TNF-α **(D)**, IL-6 **(E)**, and IL-17 **(F)** levels in the serum of rats were determined by ELISA. Tn-H, high-dose THLs group; Tn-L, low-dose THLs group. ^**^p < 0.01 vs. OVX group; ^**^p < 0.01 vs. OVX group.

LEfSe analysis was used to confirm the significant change of the microbiota. LDA score (Figure 4A) showed that there were 16 species of bacteria with statistical difference between the groups, and the specific bacteria in OVX group were *o_Bacteroidales, c_Bacteroidia*, and *p_Bacteroidete*, which indicated that *Bacteroidetes* might be correlated with the pathogenesis of bone loss. After high-dose THLs treatment, *o_Erysipelotrichales, f_Erysipelotrichaceae, c_Erysipelotrichia, g_Blautia*, and *f_Carnobacteriaceae* had significant changes. It is speculated that these bacteria were related to the improvement of bone loss. All the results presented above indicated that the gut microbiota composition of OVX rats was dramatically changed after THLs treatment.

Next, the species abundances of the top 35 species of bacteria at the genus level were displayed by color densities in the heatmap (Figure 4B) and clustered according to the similarity of the abundances of each species. *Alistipes* increased significantly after the ovariectomy, which was reversed after THLs treatment. Therefore, we hypothesized that *Alistipes* is closely connected with the pathogens of bone loss. More interestingly, a variety of new bacteria increased in the treatment of THLs. In the low-dose group, *Oscillospira, Phasolarctobacterium*, and *Roseburia* were notably increased, in the high-dose group, *Fusicatenibacter, Dubosiella*, and *Blautia* were significantly increased, and *Marvinbryantia* and *Allobaculum* were also on the rise. It suggests that these bacteria may be involved in the improvement of bone loss.

Inflammation is an essential influence factor of bone loss (40). Figures 4C–F showed that the inflammatory cytokines (M-CSF, TNF-α, IL-6, and IL-17) in the serum of rats were significantly increased after the ovariectomy. However, THLs treatment significantly downregulated the expression of inflammatory cytokines. Summing up the above, all the transformations indicated that there was a necessary relationship between the improvement of bone loss by THLs and the regulation of gut microbiota. THLs reduced inflammation by preventing the colonization of *Alistipes* and further saved bone loss.

### THLs Improved Bone Loss by Altering Serum Metabolic Pathways

**Figure 5:**
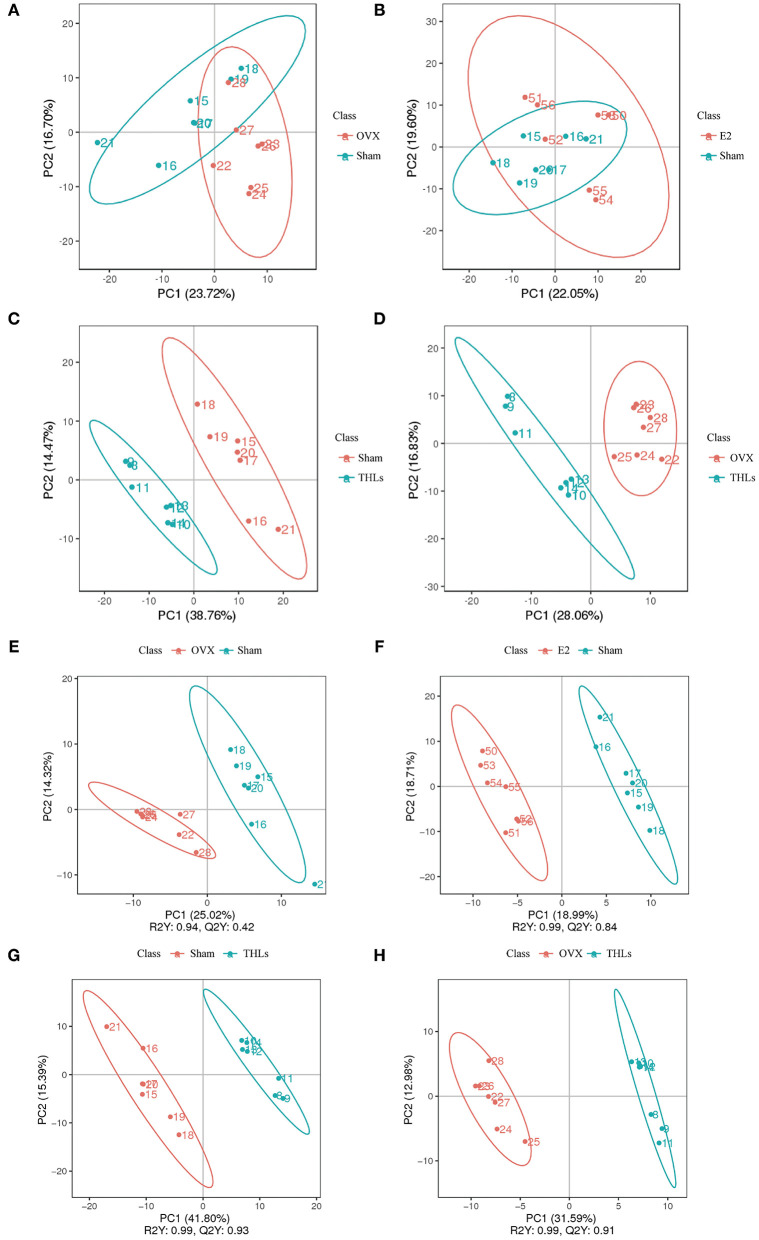
The serum metabolism of rat was changed by THLs. PCA and the partial least squares discriminant analysis (PLS-DA). **(A)** OVX vs. Sham; **(B)** E2 vs. Sham; **(C)** Sham vs. THLs; **(D)** OVX vs. THLs; **(E)** OVX vs. Sham; **(F)** E2 vs. Sham; **(G)** Sham vs. THLs; **(H)** OVX vs. THLs; the abscissa PC1 and ordinate PC2 represent the scores of the first and second principal components, respectively. R2Y represents the interpretation rate of the model, Q2Y is used to evaluate the predictive ability of PLS-DA model, and when R2Y is greater than Q2Y, it means that the model is well-established.

Principal component analysis (PCA) (Figures 5A–D) revealed that there were significant changes in serum metabolites between Sham and OVX groups. Furthermore, the composition of metabolites among THLs and OVX group was completely separated. To determine whether THLs influenced the metabolic pattern of OVX rats, the partial least squares discriminant analysis (PLS-DA) model was constructed. As we can see from Figures 5E–H, after the ovariectomy, the total types of serum metabolites in rats were greatly reduced and more concentrated. The composition of serum metabolites in E2 group was similar to Sham group. Interestingly, after THLs administration, serum metabolites were more concentrated than those in Sham group, but more dispersed than OVX group. Variable importance in the projection (VIP), fold change (FC), and *p*-value were used for screening of differential metabolites. With the setting that VIP > 1.0, FC > 1.5 or FC < 0.667, and *p* < 0.05, the screening results were displayed in Table 3, and 660 metabolites and 311 metabolites were detected in the positive and negative ion model, respectively. In conclusion, results of PCA, PLS-DA, and differential metabolites screening implied that THLs have intervened the metabolic process of OVX rats to some degree.

**Table 3:** Results of differential metabolites screened between groups.

**Compared samples**	**Num. of total ident**.	**Num. of total sig**.	**Num. of sig. up**	**Num. of sig. down**
THLs. vs. Sham_pos	660	161	71	90
THLs. vs. E2_pos	660	169	99	70
THLs. vs. OVX_pos	660	113	41	72
E2. vs. Sham_pos	660	98	20	78
E2. vs. OVX_pos	660	94	24	70
OVX. vs. Sham_pos	660	88	41	47
THLs. vs. Sham_neg	311	99	67	32
THLs. vs. E2_neg	311	127	95	32
THLs. vs. OVX_neg	311	59	29	30
E2. vs. Sham_neg	311	40	8	32
E2. vs. OVX_neg	311	69	11	58
OVX. vs. Sham_neg	311	36	28	8

**Figure 6:**
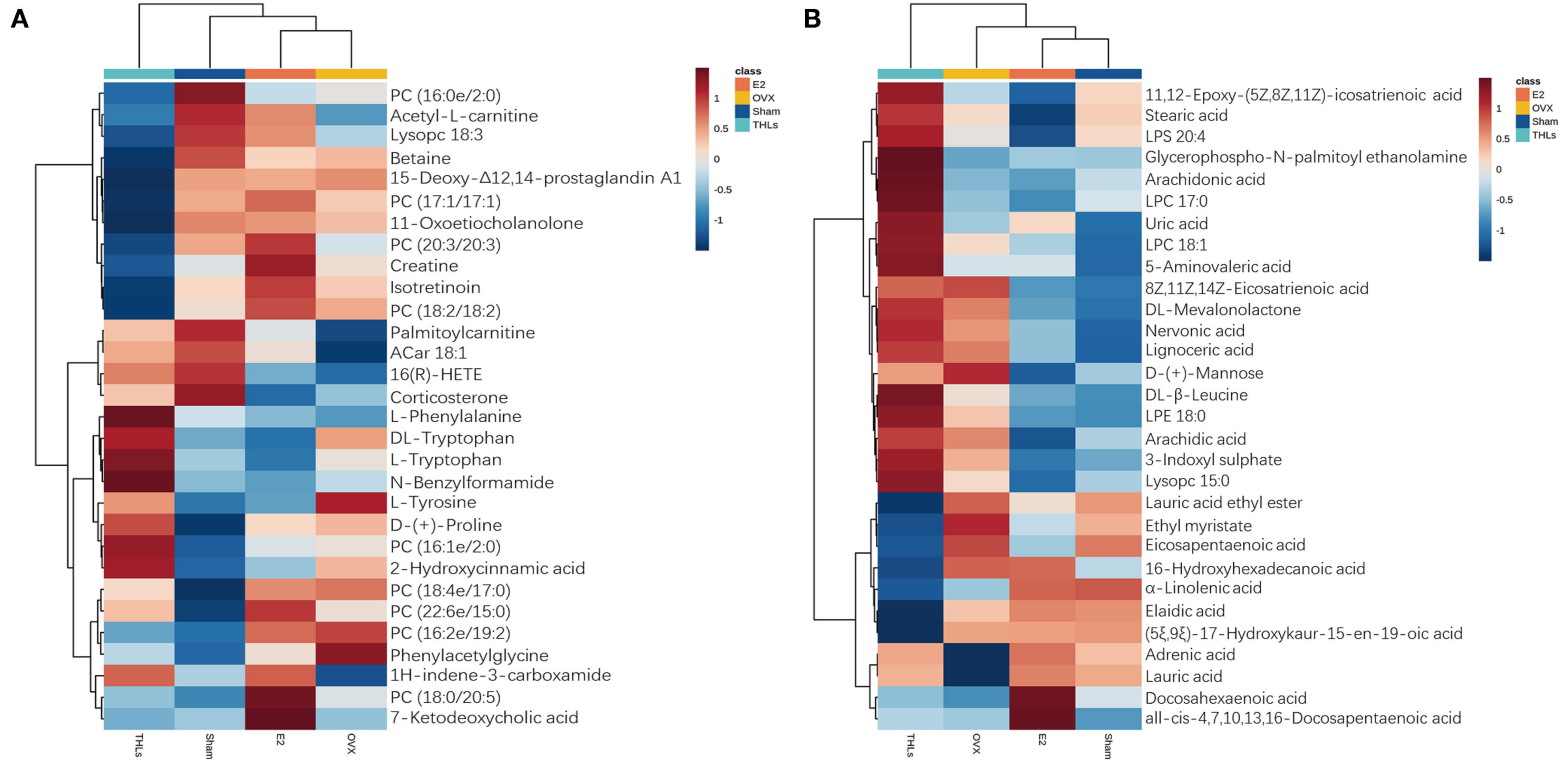
HCA for differential metabolites of top 20. **(A)** Positive ion model. **(B)** Negative ion model. Vertical is the clustering of samples, and horizontal is the clustering of metabolites. The shorter the cluster branches, the higher the similarity. The clustering relationship of metabolite content between groups can be seen *via* horizontal comparison.

To explore the potential metabolites that cause bone loss after the ovariectomy, we carried out hierarchical clustering analysis (HCA) for all differential metabolites in groups. The results stood out clearly on the Figures 6A,B, ovariectomy increased 6 metabolites [(phenylacetylglycine, L-tyrosine, D-(+)-mannose, PC (16:2e/19:2), PC (18:4e/17:0), and 8Z, 11Z, 14Z-eicosatrienoic acid] in the serum of rats. It suggested that these metabolites are closely relative to pathogenesis of bone loss. After treatment of THLs, the abnormal increase of the above metabolites was reversed. In addition, metabolites such as L-phenylalanine, N-benzylformarmide, PC (16:1e/2:0), 1H-indene-3-carboxamide, glycerophospho-N-palmitoyl ethanolamine, arachidonic acid, LPC 17:0, uric acid, and 5-aminovaleric acid were significantly increased. It indicated that these nine metabolites are presumably related to the mechanism of improving bone loss that caused by estrogen deficiency.

**Figure 7:**
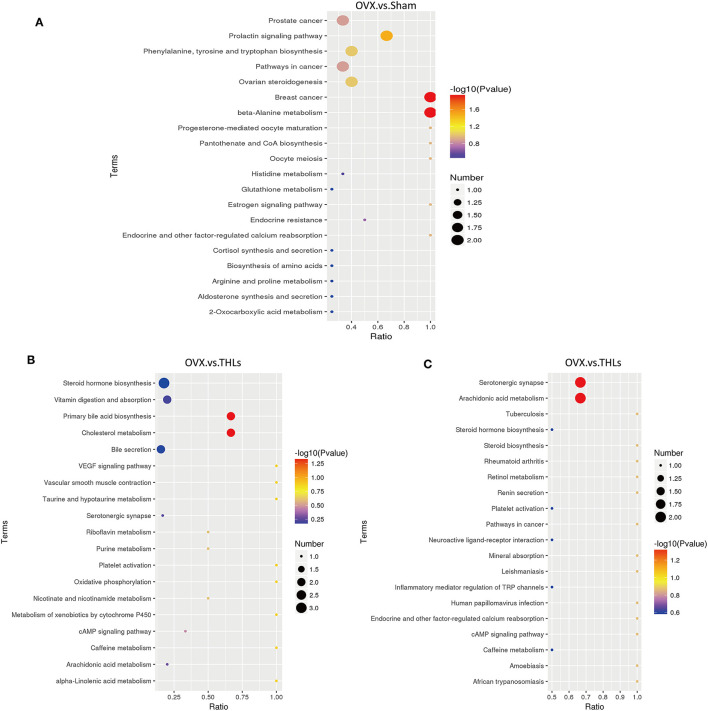
KEGG enrichment bubble maps of differential metabolites: **(A)** OVX vs. Sham in the positive ion model; **(B)** OVX vs. THLs in the positive ion model; and **(C)** OVX vs. THLs in the negative ion model. The abscissa is the ratio of the number of the differential metabolites in metabolism pathway to the number of the all metabolites in the pathway, and greater ratio represents higher degree of enrichment. The color of bubble represents *p*-value, the smaller the *p*-value, the more reliable of the test. The size of bubble represents the number of differential metabolites.

For further research on how the potential metabolic biomarkers affect bone loss, pathway enrichment can be used to look for the most critical biochemical metabolic pathways and signal transduction pathways involved in differential metabolites. The analysis results in the form of enrichment bubble pattern, and Figure 7A displayed harmful metabolites produced in serum of rats after ovariectomy mainly influence three pathways: beta-alanine metabolism, breast cancer, and prolactin signaling pathway. From Figures 7B,C, it can be seen that the main metabolic pathways for the differential metabolites between the THLs and OVX groups were participated in the regulation of arachidonic acid metabolism, serotonergic synapse, primary bile acid biosynthesis, and cholesterol metabolism. These results suggested that the above metabolic pathways may be the key pathways for THLs to protect bone. In conclusion, all results indicated that THLs improved bone loss through altering serum metabolites and metabolic pathways.

## Discussion

Osteoporosis is characterized by a remarkable decrease in bone mineral density and an increased fracture (41). Postmenopausal women lose bone rapidly, especially the trabecular bone, and the risk of fracture is higher. The reason is that postmenopausal women lack estrogen and lose the balance between bone resorption and bone formation (42, 43). This experiment used *Tilapia nilotica* head as raw material to prepare THLs and verified the improvement effect of THLs on bone loss through OVX rat model.

Composition and fatty acid analysis of THLs showed that THLs are composed of NL, PL, and GL and are rich in PUFA such as EPA and DHA. Among them, PL had the highest content of Ω-3 PUFA. A mass of evidence indicated that fish oil richly in Ω-3 PUFA is beneficial to bone health (44–47). Harries (48) found that Ω-3 PUFA is closely related to the reduction of osteoporotic fractures. Mao et al. (33) found that DHA-rich phosphatidylcholine, primary constituent of PL, has the effect of promoting bone formation. These findings are consistent with our results. In addition, Che et al. (49) proved that administered DHA enriched phospholipids and DHA-enriched triglyceride could modulate inflammatory cytokine and gut microbiota, and Gu et al. (34) also gained the results that *Tilapia nilotica* head glycolipids reduced inflammation by regulating the gut microbiota. Combined with above studies, it suggested that there is necessity to establish ovariectomized rat model for studying the effects of THLs on bone loss through gut microbiota.

Xiao et al. (50) proved that the bone mineral density of ovariectomized rats continues to decline, and the trabecular bone microstructure deteriorates, which was consistent with the Micro-CT results of the OVX group in this experiment. This experiment compared the Micro-CT data of THLs-treated rats and OVX group rats. It was proved that THLs could significantly increase Conn.D and Tb.N, decrease Tb.Sp and SMI, improve the microstructure of bone tissue, and improve the bone loss of ovariectomized rats. We measured the serum levels of bone resorption markers in rats to evaluate the bone loss. Our study proved that THLs can downregulate the level of bone resorption indexes (TRACP-5β, CTX-1, Cath-K, and MMP-9) in the serum of OVX rats. Moreover, OPG makes a critical role as a bone-protective factor by combining to RANKL and prevention of excessive bone resorption (51). Experimental results showed that THLs reversed the severe imbalance of OPG/RANKL in OVX rats and saved bone loss. To sum up, we speculated that THLs improved bone loss by decreasing the bone resorption.

Gut microbiota can be regarded as a treatment target for bone loss because that gut microbiota could conduce to normal bone health and reduce bone loss (52). Estrogen deficiencies can cause disorder of gut microbiota and influence immune system and bone resorption, resulting in osteoporosis (53). According to sequencing data, as for differences of rats in groups, the bacterial diversity of OVX rats was overtly lower than Sham rats. Besides, THLs markedly changed the constitution of the gut microbiota in OVX rats. The ratio of *Firmicutes* to *Bacteroidetes* indicates the status of metabolic disease such as osteoporosis. Compared with the Sham rats, the F/B ratio was lower in OVX rats, but the change was reversed by THLs, which make gut microbiota recover to normal status and produce certain beneficial bacteria. *Alistipes* was reported as a disease-related gut bacteria that can cause inflammation (54). Coincidently, our study observed that *Alistipes* increased markedly in OVX rats than Sham rats, which indicated *Alistipes* could bring about bone loss *via* colonization in gut. Meanwhile, compared with OVX group, beneficial bacterium such as *Oscillospira, Roseburia*, and *Dubosiella* increased dramatically. It is reported that *Oscillospira* can regulate systemic metabolic diseases such as type 2 diabetes mellitus through immune system (55). *Roseburia* and *Dubosiella* also could improve osteoporosis through recovering metabolic disorders (56). Our results indicated that gut microbiota can improve bone loss through decreasing of harmful bacteria and increasing of beneficial bacteria. To test whether above the increase of the beneficial bacteria improves bone loss through regulating inflammation and metabolism, we further measured the serum proinflammation factors and serum metabolites.

Evidences demonstrated that gut microbiota could affect the inflammation state to regulate bone mass, and the inflammation is connected with bone metabolism (21, 25, 57). The changes of M-CSF suggested that most macrophages differentiate into M1 macrophages when estrogen is deficient. M1 macrophages play proinflammatory effect by overexpressing plentiful inducible nitric oxide synthase (iNOS) and tumor necrosis factor (TNF) (58). After THLs intervention, the levels of proinflammatory factor TNF-α, IL-6, and IL-17 had decreased notably compared with OVX rats, which reduced inflammation and prevented bone resorption. Therefore, THLs reduced inflammation environment through regulating gut microbiota, thereby inhibiting bone loss.

There was evidence showed that gut microbiota is relative to the metabolic disorders (59). To further confirm that whether THLs could promote bone formation through altering the composition of metabolites in OVX rats, we measured the serum metabolism of rats with LC/MS-MS. After the ovariectomy, the composition of metabolites in rats changed significantly and reflected in the increase of phenylacetylglycine, L-tyrosine, D-(+)-mannose, PC (16:2e/19:2), PC (18:4e/17:0), and 8Z, 11Z, 14Z-eicosatrienoic acid, etc. KEGG analysis showed that these changes affected the metabolic pathways which included beta-alanine metabolism, breast cancer, and prolactin signaling pathway. Studies have found that beta-alanine metabolism was probably linked with the sclerosis of subchondral bone (60) and liver tissue damage (61). Shemanko (62) proved that prolactin had an effect on reducing the time to bone metastasis in patients with breast cancer and provoked breast cancer cell-mediated lytic osteoclast formation. Our study conformed to above discoveries and suggested that there is a correlation between the pathogenesis of bone loss and beta-alanine metabolism, prolactin signaling pathway. After treatment with THLs, increased levels of L-phenylalanine, N-benzylformarmide, PC (16:1e/2:0), 1H-indene-3-carboxamide, glycerophospho-N-palmitoyl ethanolamine, arachidonic acid, LPC 17:0, uric acid, and 5-aminovaleric acid were observed, which suggested that the regulation of bone metabolism of THLs was associated with above metabolites. Metabolites affected metabolic pathway such as arachidonic acid metabolism, serotonergic synapse, primary bile acid biosynthesis, and cholesterol metabolism. Among them, arachidonic acid metabolism affects bone resorption through inflammation (63), and arachidonic acid-rich oil had opposite effects on bone resorption in a mice model of postmenopausal osteoporosis (64). Serotonin, which is synthesized in the bone, can modulate bone metabolism (65). Study has proved that lignans can affect postmenopausal osteoporosis through primary bile acid biosynthesis (66). It is reported that cholesterol content positively associated with bone mineral density of postmenopausal women (67). Thus, THLs could putatively exert a major metabolic effect on improving bone loss through arachidonic acid metabolism, serotonergic synapse, primary bile acid biosynthesis, and cholesterol metabolism.

What is worth discussing is that we notice that the bone loss improving effect of low-dose THLs was better than high-dose THLs. The reason may be the excess lipids in high-dose THLs damaged to bone. We have proved neutral lipids accounted for 77.84% in THLs, and the most component of neutral lipids is triglyceride. In recent years, numerous studies explored the relationship between high-fat diet to bone damage. Wang et al. (68) established hyperlipidemia rat model, which confirmed that high-fat diet has harmful effect to bone microstructure. The study of Zhang et al. (69) was also based on the theory that high-fat diet can induce bone loss. Hence, it is reasonable to consider that high-dose THLs improved the bone poorly because of excess lipids.

## Conclusion

It has been experimentally verified that the regulation of the gut microbiota plays a key role in bone loss. THLs can improve bone microstructure and bone homeostasis in the OVX rats. 16S rDNA sequencing analysis showed that THLs may rescue bone loss through regulating gut microbiota and inhibiting the colonization of *Alistipes*. Inflammation analysis and serum metabolism further elucidated the gut microbiota played an important role in improving bone loss through regulating the inflammation and metabolic pathways. In short, THLs can improve the bone loss in ovariectomized rats. The results of this study promoted the high-value utilization of fish by-products and provided a new thought for the development of natural active ingredients for improving bone loss.

## Data Availability Statement

The datasets presented in this study can be found in online repositories. The names of the repository/repositories and accession number(s) can be found at: https://magic.novogene.com, X101SC20051435-Z01-F003-B1-41.

## Ethics Statement

The animal study was reviewed and approved by Hainan University Institutional Animal Use and Care Committee (HNDX2020072).

## Author Contributions

Project administration, methodology, software, validation, formal analysis, data curation, and writing—original draft preparation were performed by YZ, SL, and FM. Conceptualization, investigation, writing—reviewing and editing were done by MZ. Visualization, supervision, funding acquisition, and resources were conceived by GX and XS. All authors have read and agreed to the published version of the manuscript.

## Funding

This work was supported by the National Key R&D Programs of China (Number 2018YFD0901103), Hainan Provincial Natural Science Foundation of China (Number 2019RC093), and the Program of Hainan Association for Science and Technology Plans to Youth R&D Innovation (Number QCXM202003).

## Conflict of Interest

The authors declare that the research was conducted in the absence of any commercial or financial relationships that could be construed as a potential conflict of interest.

## Publisher's Note

All claims expressed in this article are solely those of the authors and do not necessarily represent those of their affiliated organizations, or those of the publisher, the editors and the reviewers. Any product that may be evaluated in this article, or claim that may be made by its manufacturer, is not guaranteed or endorsed by the publisher.
